# Phosphodiesterase 4 regulates pyroptosis in subarachnoid hemorrhage

**DOI:** 10.4103/NRR.NRR-D-24-01381

**Published:** 2025-06-19

**Authors:** Jiahe Tan, Yinrui Ma, Rui Song, Hongjiang Ye, Jun Su, Zhaohui He

**Affiliations:** 1 Department of Neurosurgery, The First Affiliated Hospital of Chongqing Medical University, Chongqing, China; 2 Department of Anesthesiology, The Second Affiliated Hospital of Chongqing Medical University, Chongqing, China

**Keywords:** early brain injury, etazolate, lysosome function, mitochondrial function, neuron, nucleotide-binding oligomerization domain-like receptor pyrin domain containing 3 (NLRP3), nuclear factor kappa-B, phosphodiesterase 4, pyroptosis, subarachnoid hemorrhage

## Abstract

Phosphodiesterase 4 is a key enzyme involved in the regulation of cell signal transduction, but its role in subarachnoid hemorrhage remains unclear. Neuronal pyroptosis has been reported to be involved in early brain injury after subarachnoid hemorrhage. This study aimed to investigate whether phosphodiesterase 4 contributes to early brain injury after subarachnoid hemorrhage by mediating neuronal pyroptosis and its related mechanisms. Endovascular perforation of male C57BL/6J mice was performed to model subarachnoid hemorrhage *in vivo*, and oxyhemoglobin was added to the culture medium of primary neurons to model subarachnoid hemorrhage *in vitro*. A phosphodiesterase 4-specific inhibitor, etazolate, was intraperitoneally injected 30 minutes after subarachnoid hemorrhage induction. Small interfering RNA (siRNA) was administered intracerebroventricularly 72 hours before subarachnoid hemorrhage to achieve genetic knockdown of phosphodiesterase 4. To investigate the mechanism, a nucleotide-binding oligomerization domain-like receptor pyrin domain containing 3 (NLRP3)-specific agonist, nigericin, was intracerebroventricularly injected 60 minutes before subarachnoid hemorrhage. Neuronal phosphodiesterase 4 expression increased after subarachnoid hemorrhage and reached the highest point at 24 hours. Etazolate treatment reduced neurological deficits and brain edema in mice, alleviated neuronal pyroptosis and inflammatory response, and improved neuronal injury. Treatment with phosphodiesterase 4 siRNA had the same neuroprotective effects as etazolate. Mechanistically, phosphodiesterase 4 triggered the nuclear factor kappa-B pathway, and simultaneously caused lysosomal and mitochondrial dysfunction after subarachnoid hemorrhage, which promoted NLRP3 inflammasome activation and induced neuronal pyroptosis. Blocking of phosphodiesterase 4 inhibited the nuclear factor kappa-B pathway, and improved lysosome and mitochondrial function. Activation of NLRP3 reversed the neuroprotective effects of etazolate without affecting phosphodiesterase 4 expression. Together, the results indicate that phosphodiesterase 4 regulates NLRP3-mediated neuronal pyroptosis in early brain injury after subarachnoid hemorrhage. Phosphodiesterase 4 may be a potential therapeutic molecular target for subarachnoid hemorrhage.

## Graphical abstract

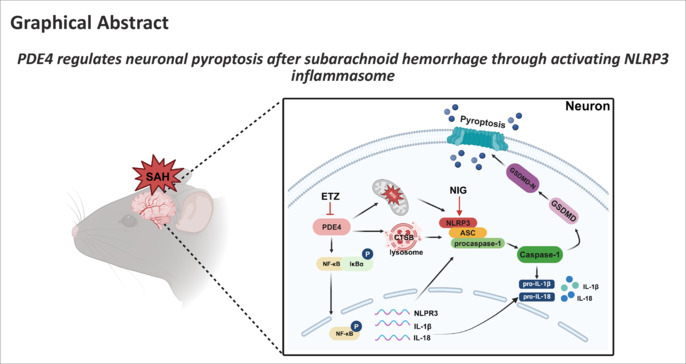

## Introduction

Subarachnoid hemorrhage (SAH) is a common and severe cerebrovascular disease with extremely high disability and mortality rates (Macdonald and Schweizer, 2017). Early brain injury (EBI) refers to a series of direct brain injuries occurring within 72 hours after SAH, and has been identified as a crucial contributor to the unfavorable outcomes of SAH (Cahill et al., 2006; Rass and Helbok, 2019). Pyroptosis is a form of programmed cell death marked by persistent cell swelling that causes cell membranes to burst, releasing cellular contents and triggering a robust inflammatory reaction (Yu et al., 2021; Zhang et al., 2024; Zheng et al., 2025). Nucleotide-binding oligomerization domain-like receptor pyrin domain containing 3 (NLRP3) inflammasome is a key molecule in regulating the classical pathway of pyroptosis (Coll et al., 2022). A growing number of studies indicate that neuronal pyroptosis significantly contributes to the pathology of EBI, although the exact mechanisms remain unclear (Yuan et al., 2020; Fang et al., 2022; Liu et al., 2023). Therefore, exploring new molecular targets and therapeutic drugs for regulating neuronal pyroptosis is particularly critical to improve EBI after SAH.

Phosphodiesterase 4 (PDE4) is an important member of the phosphodiesterase family that specifically breaks down cyclic adenosine monophosphate (cAMP), causing changes in cAMP concentration that trigger downstream cascades, and it is closely related to various diseases (Gancedo, 2013; Fertig and Baillie, 2018). PDE4 is expressed in neurons, and inhibition of PDE4 through pharmacological action alleviates neuronal apoptosis of SAH in rats by boosting the sirtuin1 level and enhancing Akt phosphorylation (Li et al., 2018). Studies have shown that PDE4 inhibitors have neuroprotective effects after SAH, but the exact mechanisms are not fully understood (Wu et al., 2017; Li et al., 2018). At present, no studies have confirmed the regulatory relationship and related mechanisms between PDE4 and NLRP3 or neuronal pyroptosis.

Using animal and cell models of SAH, this study aimed to clarify whether PDE4 is involved in EBI through the mediation of neuronal pyroptosis, and whether inhibiting PDE4 has neuroprotective effects through corresponding mechanisms. The findings provide experimental evidence and a clinical translation direction in the effort to identify new treatment options for neurological dysfunction after SAH.

## Methods

### Animals

Only male mice were used in this study to avoid the influence of periodic hormonal fluctuations in female mice on the results of the experiments (Dinh et al., 2024). A total of 335 adult male C57BL/6J mice (8–10 weeks old, 22–25 g) were purchased and housed at the Laboratory Animal Center of Chongqing Medical University, China (license No. SCXK (Yu) 2022-0010)). The mice were allowed to eat freely (sterile feed and drinking water), and the cages underwent a 12-hour light-dark cycle to simulate the circadian rhythm. The temperature and relative humidity were controlled at approximately 22°C and 60%, respectively. All mouse experiments were conducted in accordance with the National Institutes of Health guidelines for the Care and Use of Laboratory Animals (8^th^ ed., National Research Council, 2011). The experimental protocols were approved by the Laboratory Animal Management and Use Committee of Chongqing Medical University on September 5, 2023 (approval No. IACUC-CQMU-2023-0235).

### Study design

#### Experiment 1

Mice were randomly divided into six groups to determine PDE4’s involvement in EBI and identify the changes of PDE4 after SAH: Sham (sham surgery), 6, 12, 24, 48, and 72 hours after SAH (*n* = 6 per group). The modified Garcia score, balance beam test, Rotarod test, and brain water content measurement were used to assess the neurological function and brain edema of mice. Western blot analysis was performed to detect PDE4 expression in the cortex of the frontal and temporal lobes (hereafter referred to as cortex). Four extra mice from the Sham group and SAH 24 hours group were used to perform confocal immunofluorescence (IF) (*n* = 2 per group).

#### Experiment 2

A PDE4-specific inhibitor, etazolate (ETZ; 1 mg/mL diluted with saline, Cat# B6304, APExBIO, Houston, TX, USA) was intraperitoneally (i.p.) injected 30 minutes after SAH to investigate the influence of PDE4 on EBI. Saline (Vehicle, the same volume as ETZ) was i.p. injected 30 minutes after SAH as a control. Mice were randomly divided into five groups: Sham + Vehicle, SAH + Vehicle, SAH + ETZ (5 mg/kg), SAH + ETZ (10 mg/kg), and SAH + ETZ (15 mg/kg) (*n* = 12 per group). The modified Garcia score, balance beam test, Rotarod test, and brain water content measurement were used to assess the neurological function and brain edema of mice. Western blot analysis was conducted to determine PDE4 levels in mice cortex.

#### Experiment 3

On the basis of the findings from Experiment 2, a dosage of 10 mg/kg ETZ was used to investigate the impact of PDE4 on pyroptosis and its corresponding mechanisms. The experimental procedures were performed as described in Experiment 2. Mice were randomly divided into three groups: Sham + Vehicle, SAH + Vehicle, and SAH + ETZ (*n* = 24 per group). Transmission electron microscopy (TEM), IF, western blot, enzyme-linked immunosorbent assay (ELISA), Fluoro-Jade C (FJC) staining, hematoxylin-eosin (HE) staining, Nissl staining, and flow cytometry (reactive oxygen species [ROS] measurement) were used.

#### Experiment 4

To investigate the impact of genetic suppression of PDE4 on pyroptosis, 3 µL PDE4 small interfering RNA (siRNA) and scrambled siRNA (Beijing Tsingke, China) were administered intracerebroventricularly (i.c.v.) 72 hours before SAH. Mice were randomly divided into four groups: Sham, SAH, SAH + Scr siRNA, and SAH + PDE4 siRNA (*n* = 12 per group). Assessment methods used were the modified Garcia score, balance beam test, Rotarod test, brain water content measurement, IF, western blot, and ELISA.

#### Experiment 5

To determine whether PDE4 influences pyroptosis through NLRP3 regulation, 2 µL nigericin (NIG; 2 μg/μL diluted with saline, N1495, Thermo Fisher Scientific, Waltham, MA, USA), an NLRP3-specific agonist, was i.c.v. administered 60 minutes before SAH. Mice were randomly divided into four groups: Sham + Vehicle, SAH + Vehicle, SAH + ETZ, and SAH + ETZ + NIG (*n* = 12 per group). The modified Garcia score, balance beam test, Rotarod test, and brain water content measurement were used to assess the neurological function and brain edema of mice. Western blot analysis and ELISA were conducted to identify pyroptosis-related molecules levels in mouse cortex.

**Figure 1:**
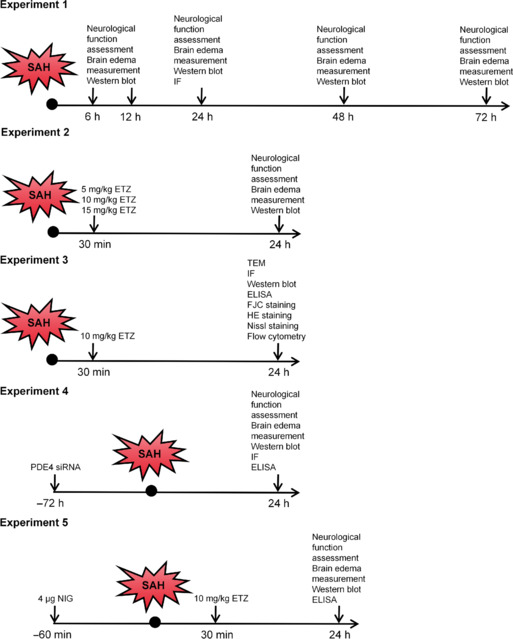
Experimental design diagram. ELISA: Enzyme linked immunosorbent assay; ETZ: etazolate; FJC: Fluoro-Jade C; HE: hematoxylin-eosin; IF: immunofluorescence; NIG: nigericin; PDE4: phosphodiesterase 4; SAH: subarachnoid hemorrhage; siRNA: small interfering RNA; TEM: transmission electron microscopy.

The experimental flowchart and experimental design diagram are shown in **Figure 1**.

### Mouse subarachnoid hemorrhage model

An endovascular perforation technique was used to create the mouse model of SAH, as described previously (Liu et al., 2017). In brief, the mice were anesthetized by i.p. injection of 1% pentobarbital (50 mg/kg, diluted with saline, P3761, Sigma). Then, the left carotid artery and its branches were exposed from the tissue. A pointed puncture nylon (1620A2, Beijing Cinontech, China) was inserted into the internal carotid artery through the stump of the external carotid artery. The nylon was continuously pushed forward after feeling resistance to puncture the junction of the anterior and middle cerebral artery. The Sham group underwent an identical process, excluding the artery perforation.

### Cell culture and *in vitro* subarachnoid hemorrhage model

Primary neurons were extracted and cultured using previous methods with modifications (Tao et al., 2019). Mice were euthanized by rapid freezing with liquid nitrogen at postnatal days 0–2, and brains were dissected and placed in cold phosphate buffered saline (PBS) with 1% antibiotics. Then, brains were transferred to a sterile dish, meninges were removed, and cortices were dissected out. Next, the tissue was finely minced, incubated with 0.25% trypsin for 15 minutes at 37°C, neutralized with culture medium (DMEM/F12, 21331220, Gibco, Carlsbad, CA, USA), and gently triturated with a pipette. The suspension was passed through a 40-µm cell strainer to eliminate debris. The isolated cells were then cultured at a density of 1 × 10^6^ cells/cm^2^ on poly-D-lysine-coated plates and incubated with 5% CO_2_ at 37°C. The growth medium (Neurobasal, 21103049 with B27, 17504044, Gibco) and antibiotics were changed every 2 or 3 days while maintaining incubation conditions.

In accordance with previous methods (Chen et al., 2020), oxyhemoglobin (OxyHb, H2625, Sigma) was used to simulate the external environment of primary neurons after SAH to establish an *in vitro* SAH model. The growth medium was changed to one with 60% OxyHb concentration in the OxyHb + Vehicle and OxyHb + ETZ groups, whereas the Control + Vehicle group was treated with ordinary growth medium exchange. In accordance with the instructions and a previous study (Marcade et al., 2008), primary neurons were treated with ETZ (1 μM, diluted with DMSO, D8371, Solarbio, Beijing, China) 30 minutes after being exposed to OxyHb in the OxyHb + ETZ group, and the other two groups were treated with the same volume of DMSO.

### Intracerebroventricular injection

As described above, the mice were anesthetized with 1% pentobarbital. Then, a Hamilton syringe (Hamilton, Bonaduz, Switzerland) with a volume of 10 μL was positioned into the left lateral ventricle using the coordinates 0.4 mm posterior and 1.0 mm lateral to the bregma, and 3.0 mm beneath the dura mater (the coordinates were confirmed according to the Paxinos and Franklin’s the Mouse Brain in Stereotaxic Coordinates and previous research) (Paxinos and Franklin, 2013; Nishikawa et al., 2018). Then, 2 μL of normal saline or 4 μg of NIG dissolved in 2 μL normal saline was administered 60 minutes before SAH (Li et al., 2020). For the genetic knockdown experiments, 3 μL of PDE4 siRNA (sense: 5ʹ-GGA GGA CCU CUU AGC ACA AGA-3ʹ; antisense: 5ʹ-UUG UGC UAA GAG GUC CUC CUG-3ʹ) or Scr siRNA (designed and prepared by Beijing Tsingke, China) was administered 72 hours before SAH.

### Subarachnoid hemorrhage grade

SAH severity was assessed using the method proposed by Sugawara et al. for SAH animal model classification (Sugawara et al., 2008) by a researcher blinded to the animal groupings. At 24 hours following SAH, the basal cistern of each mouse was divided into six parts: the pons was divided into two parts by the midpoint horizontal line, and the two parts of the bilateral cerebral hemispheres were divided into four parts by the midpoint horizontal line of the circle of Willis. The degree of bleeding in each part was scored (0–3), and the overall score was the aggregate of these ratings (0–18). Mice with mild SAH (0–7) were excluded, and only mice with moderate (8–12) or severe SAH (13–18) were used for subsequent experiments.

### Neurological function assessment

The neurological function of mice was assessed using the modified Garcia score, balance beam test, and Rotarod test by a researcher blinded to the animal groupings. The modified Garcia score comprised scores for six components, which were summed for the total score (3–18), with lower scores indicating worse neurological function (Sugawara et al., 2008). In the balance beam test, mice were placed on one side of the beam (Ugo Basile, Milan, Italy) and observed for 1 minute. Their walking posture was evaluated, and the time of falling was recorded. The scoring criteria (0–6) were based on the study by Luong et al. (2011), with higher scores indicating worse neurological function. For the Rotarod test, mice were introduced to the device (Ugo Basile) and given time to get used to the stationary bar before encountering the rotating axis. The initial speed was set at 4.5 rotations per minute (rpm), and the speed was gradually increased to 8 rpm over a 2-minute period. The time of mice falling was recorded, with shorter times indicating worse neurological function (Choi et al., 2021).

### Brain edema measurement

In accordance with previous methods (Xie et al., 2017), the brains of mice were harvested 24 hours after SAH. Then, the cerebellum and brainstem were removed, and the remaining tissue was immediately weighed to obtain the wet weight. The tissue was weighed again after drying in an oven for 48 hours at 60°C to obtain the dry weight. The brain water content (%) was determined using the formula: (wet weight – dry weight) / wet weight × 100.

### Immunofluorescence and Fluoro-Jade C staining

IF staining was performed on brain paraffin sections that were deparaffinized by heating at 60°C for 1 hour, and rehydrated via graded alcohols and water. Antigens were retrieved by incubation in citric acid buffer (pH 6.0, P0081, Beyotime, Beijing, China) for 20 minutes at 95°C and then cooling. To prevent nonspecific binding, sections were treated with 10% goat serum (C0265, Beyotime) for 1 hour. Afterward, the tissue was incubated with the primary antibody at 4°C overnight, followed by rinsing with PBS. Fluorophore-conjugated secondary antibody was incubated for 1 hour at approximately 22°C, with protection from light, followed by PBS washes. Sections were mounted with antifade reagent with 4′,6-diamidino-2-phenylindole (P0131, Beyotime), covered with a coverslip, and examined under the fluorescence microscope (LSM780, Zeiss, Germany). For IF staining of primary neurons, cells were fixed in 4% paraformaldehyde (PFA) for 10 minutes, and then the same steps as above were followed (Fu et al., 2024). The primary antibodies used were as follows: rabbit anti-PDE4 (1:100, Cat# ab14628, Abcam, Cambridge, UK), rabbit anti-NLRP3 (1:200, MA5-32255, Thermo Fisher Scientific), rabbit anti-Caspase-1 (1:200, Cat# PA5-87536, Thermo Fisher Scientific), rabbit anti-gasdermin D (GSDMD; 1:200, Cat# PA5-116815, Thermo Fisher Scientific), rabbit anti-lysosomal associated membrane protein 1 (LAMP11; 1:200, Cat# ab24170, Abcam), rabbit anti-cathepsin B (CTSB; 1:200, Cat# 31718, Cell Signaling Technology, Danvers, MA, USA), mouse anti-neuron-specific nuclear protein (NeuN; 1:200, Cat# ab104224, Abcam), mouse anti-ionized calcium-binding adapter molecule 1 (Iba-1; 1:200, Cat# MA5-27726, Thermo Fisher Scientific), mouse anti-glial fibrillary acidic protein (GFAP; 1:200, Cat# 60190-1-Ig, Proteintech, Wuhan, China), and mouse anti-microtubule associated protein 2 (MAP2; 1:100, Cat# 13-1500, Thermo Fisher Scientific). The secondary antibodies used were Cy3-conjugated goat anti-rabbit IgG (1:100, Cat# SA00009-2, Proteintech) and Alexa Fluor 488-conjugated goat anti-mouse IgG (1:100, Cat# ab150113, Abcam).

FJC staining was performed following the instructions of the FJC kit (TR-100-FJT, Biosensis, Thebarton, Australia) (Li et al., 2024). Brain sections were fixed with 4% PFA for 20 minutes, washed with distilled water, and subsequently exposed to 0.06% potassium permanganate for 10 minutes. Afterward, they were immersed in a 0.1% FJC solution at approximately 22°C for 30 minutes, mounted using an antifade agent, and topped with a coverslip. FJC-positive neurons, which showed clear green fluorescence, were visualized under the fluorescence microscope and counted in number per mm^2^.

For hematoxylin-eosin staining, brain sections were deparaffinized by heating at 60°C and rehydrated via graded alcohols and water. Then, the sections were stained with hematoxylin (C0105S-1, Beyotime) for 5 minutes, followed by a thorough water rinse, differentiated in acid alcohol, and rinsed again. Next, they were counterstained with eosin (C0105S-2, Beyotime) for 1 or 2 minutes, rinsed, and dehydrated via graded alcohols. Subsequently, the sections were immersed in xylene and covered with a coverslip, and examined under a light microscope (Primostar 1, Zeiss, Oberkochen, Germany) to observe denatured neurons.

For Nissl staining, brain slices were deparaffinized by heating at 60°C, washed with distilled water, and then soaked in 1% aqueous cresyl violet solution (Cat# C9140, Solarbio) for 10 minutes. Subsequently, they were washed in purified water, and then differentiated in 95% ethanol, and finally dehydrated using a series of graded alcohol solutions. Afterward, they were immersed in xylene and covered with a coverslip, and visualized under the light microscope to observe Nissl-stained cell bodies (Tao et al., 2023).

The region of interest selected for image capture was the left cortex. The positive cells and fluorescence density were counted, quantified, and analyzed using ImageJ software (version 2.14.0, National Institutes of Health, Bethesda, MD, USA) by a researcher blinded to the groupings.

### Transmission electron microscopy

The brain cortex was cut into a 1 mm^3^ sample, which was then preserved and stabilized in 2.5% glutaraldehyde and 1% osmium tetroxide, respectively. Next, the tissue underwent dehydration in graded ethanol concentrations and was encased in epoxy resin. Sections of 50–70 nm thickness were sliced by an ultramicrotome (EM KMR2, Leica, Weztlar, Germany), mounted on copper grids, treated with uranyl acetate and lead citrate stains (Chuandong Chemical), and examined using the transmission electron microscope (HT7800, Hitachi, Tokyo, Japan).

### Enzyme-linked immunosorbent assay

The inflammatory factors of the brain cortex and primary neurons were measured using a mouse interleukin (IL)-1β ELISA kit (RK04878, Abclonal, Wuhan, China) and a mouse IL-18 ELISA kit (RK00104, Abclonal) following the manufacturer’s instructions. The antigen (100 µL/well, 5 µg/mL) was first coated onto a microplate and incubated for 2 hours at 37°C. After blocking with 200 µL of 5% bovine serum albumin in PBS for 1 hour, the plate was incubated with a primary antibody (RK04878 or RK00104, Abclonal, 100 µL/well, 3 µg/mL) for 1 hour at 37°C. A secondary horseradish peroxidase (HRP)-conjugated antibody (RK04878 or RK00104, Abclonal, 100 µL/well, 1:5000 dilution) was then added and incubated for 1 hour at 37°C. Next, 100 µL of chromogenic substrate was added, and the color developed for 20 minutes in the dark. The reaction was stopped with 50 µL of stop buffer, and the absorbance was measured at 450 nm using a microplate reader (DTX-880, Beckman, Brea, CA, USA).

### Measurement of reactive oxygen species

DCFH-DA Kit (S0033S, Beyotime) was used to detect the ROS level in primary neurons. DCFH-DA was diluted to a concentration of 10 μM with DMEM/F12 medium. It was then added to the primary neurons and incubated for 20 minutes at 37°C while being protected from light. The primary neurons were examined under the fluorescence microscope after removing the medium. The fluorescence density was analyzed using ImageJ software.

The levels of total cellular or mitochondrion-specific ROS were detected with the DCFH-DA Kit (S0033S, Beyotime) or MitoSOX Green kit (M36005, Thermo Fisher Scientific) (Huang et al., 2024), according to the manufacturers’ instructions. First, 5 × 10^5^ cells were collected, then mixed with 500 μL DCFH-DA (10 μM) or MitoSOX Green working solution (5 μM), and incubated at 37°C for 30 minutes in the dark. Next, the primary neurons underwent two PBS washes, were resuspended, and were analyzed by flow cytometry (CytoFLEX, Beckman) via FlowJo software (version 10.3.0, BD Biosciences, Franklin Lake, NJ, USA).

### Western blotting

For western blot, the proteins of the brain cortex and primary neurons were extracted by conventional methods. The protein samples underwent sodium dodecyl sulfate-polyacrylamide gel electrophoresis separation and were transferred onto a polyvinylidene fluoride membrane (IPVH00010, Millipore, Bedford, MA, USA). Afterward, they were blocked with 5% milk in TBST for 1 hour, then underwent incubation with the primary antibody at 4°C overnight, followed by subsequent rinsing with TBST. HRP-conjugated secondary antibody was incubated for 1 hour at approximately 22°C, followed by TBST washes. Finally, the membrane was developed using an enhanced chemiluminescence substrate. The signal was captured with the chemiluminescence imaging system (ChemiDoc XRS+, Bio-Rad, Hercules, CA, USA), and the bands were analyzed using ImageJ software. The primary antibodies used were as follows: rabbit anti-PDE4 (1:500, ab14628, Abcam), rabbit anti-NLRP3 (1:1000, MA5-32255, Thermo Fisher Scientific), rabbit anti-apoptosis-associated speck-like protein containing a caspase recruitment domain (ASC; 1:500, PA5-50915, Thermo Fisher Scientific), rabbit anti-Caspase-1 (1:500, PA5-87536, Thermo Fisher Scientific), rabbit anti-GSDMD (1:1000, PA5-116815, Thermo Fisher Scientific), rabbit anti-phospho-inhibitor kappa B alpha (IκBα; 1:500, 2859, Cell Signaling Technology), rabbit anti-IκBα (1:1000, 9242, Cell Signaling Technology), rabbit anti-phospho-nuclear factor-kappa B (NF-κB) p65 (1:500, 3033, Cell Signaling Technology), rabbit anti-NF-κB p65 (1:1000, 8242, Cell Signaling Technology), rabbit anti-LAMP1 (1:1000, ab24170, Abcam), rabbit anti-CTSB (1:1000, 31718, Cell Signaling Technology), and mouse anti-β-actin (1:1000, 3700, Cell Signaling Technology). The secondary antibodies used were HRP-conjugated anti-rabbit IgG (1:5000, 7074, Cell Signaling Technology), and HRP-conjugated anti-mouse IgG (1:5000, 7076, Cell Signaling Technology).

### Statistical analysis

All data were analyzed statistically using GraphPad Prism software (version 9.4.1, GraphPad Software, San Diego, CA, USA, www.graphpad.com) and presented as mean ± standard deviation. The Shapiro–Wilk test was used to assess the data’s normality. When it followed a normal distribution, the one-way analysis of variance and Tukey’s *post hoc* test were used to determine differences between groups. When the data deviated from a normal distribution, the Brown–Forsythe test was used for analyzing differences between groups. *P* < 0.05 was considered statistically significant.

## Results

### Mortality, typical skull base images, and subarachnoid hemorrhage severity

**Figure 2:**
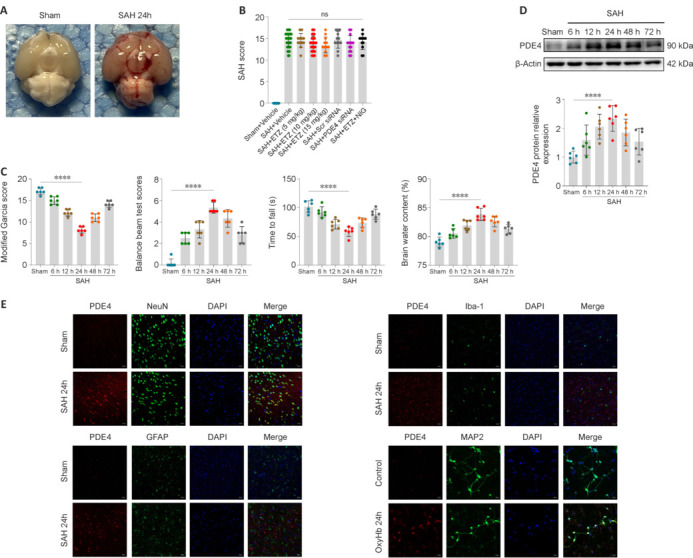
Animal experiment groups with mortality, skull base images, and SAH severity, changes in EBI severity, and PDE4 expression and cellular location. (A) Typical skull base images of Sham mice and mice 24 hours after SAH. (B) SAH severity of all SAH groups (*n* = 60, 60, 12, 48, 12, 12, 14, 12, respectively). (C) The modified Garcia score, balance beam test, Rotarod test, and brain water content of all groups in the time course experiment (6, 12, 24, 48, 72 hours after SAH). (D) Representative western blot band and relative protein expression of PDE4 in time course experiment (6, 12, 24, 48, 72 hours after SAH). (E) Confocal IF staining of PDE4 (red) with neurons (NeuN, green), microglia (Iba-1, green), astrocytes (GFAP, green), and primary neurons (MAP2, green) at 24 hours after SAH. Scale bars: 20 μm. Data are presented as mean ± SD; *n* = 6 per group except Figure 1B. ****P < 0.0001 (one-way analysis of variance with Tukey’s *post hoc* test or the Brown–Forsythe test). EBI: Early brain injury; ETZ: etazolate; IF: immunofluorescence; NIG: nigericin; ns: not significant; OxyHb: oxyhemoglobin; PDE4: phosphodiesterase 4; SAH: subarachnoid hemorrhage; Scr: scrambled; Sham: sham surgery; siRNA: small interfering RNA.

We first performed experiments to ensure uniformity of the SAH model and avoid the confounding of results caused by different degrees of hemorrhage. This study involved a total of 335 mice, with 72 in the Sham group and 263 in the SAH group. In the SAH group, the mouse mortality was 15.21% (40/263), whereas there were no deaths in the Sham group. Additionally, 17 mice with mild SAH were excluded (**Table 1**). Typical skull base images showed obvious hemorrhage in mice after successful SAH modeling (**Figure 2A**). No significant differences in mortality and SAH severity were observed between the experimental groups (**Figure 2B**).

**Table 1:** Mortality and exclusion of mice

Group	Mortality	Excluded
**Experiment 1**		
Sham	0% (0/12)	0
SAH (6, 12, 24, 48, 72 h)	14.89% (7/47)	4
**Experiment 2**		
Sham+Vehicle	0% (0/12)	0
SAH+Vehicle	14.29% (2/14)	0
SAH+ETZ (5 mg/kg)	14.29% (2/14)	0
SAH+ETZ (10 mg/kg)	13.33% (2/15)	1
SAH+ETZ (15 mg/kg)	31.58% (6/19)	1
**Experiment 3**		
Sham+Vehicle	0% (0/24)	0
SAH+Vehicle	13.33% (4/30)	2
SAH+ETZ	10.34% (3/29)	2
**Experiment 4**		
Sham	0% (0/12)	0
SAH	13.33% (2/15)	1
SAH+Scr siRNA	13.33% (2/15)	1
SAH+PDE4 siRNA	12.50% (2/16)	0
**Experiment 5**		
Sham+Vehicle	0% (0/12)	0
SAH+Vehicle	13.33% (2/15)	1
SAH+ETZ	12.50% (2/16)	2
SAH+ETZ+NIG	22.22% (4/18)	2

**Total**		
Sham	0% (0/72)	0
SAH	15.21% (40/263)	17

ETZ: Etazolate; NIG: nigericin; SAH: subarachnoid hemorrhage; Scr: scrambled; Sham: sham surgery; siRNA: small interfering RNA.

### Changes in neurological function, brain edema, and phosphodiesterase 4 expression

PDE4 involvement in EBI was investigated next. The modified Garcia score indicated an initial decline followed by an increase, reaching its lowest point at 24 hours. In the balance beam test, scores initially rose and then fell, peaking at 24 hours. The Rotarod test showed that the duration until falling first shortened and then lengthened, with the shortest duration at 24 hours. The brain water content first increased and then decreased, with the highest level at 24 hours (**Figure 2C**). Regarding PDE4 expression, the western blot analysis indicated that PDE4 protein levels initially rose and then fell, peaking at 24 hours after SAH (**Figure 2D**). The time point of the greatest PDE4 changes coincided with the peak time of EBI-related changes (24 hours after SAH), and thus it is reasonable to speculate that PDE4 may be involved in the process of EBI by increasing its expression. Therefore, the time point of subsequent experiments was selected as 24 hours after SAH.

### Cellular location of phosphodiesterase 4

Neural cells for subsequent experiments were identified by PDE4 localization. Confocal IF showed that PDE4 was localized in neurons, but not in microglia or astrocytes, and its expression increased in neurons after SAH. Similarly, PDE4 was localized in the primary cortical neurons cultured *in vitro*, and its expression increased after OxyHb induction (**Figure 2E**). Therefore, we determined that the cells of interest for the subsequent experiments were neurons.

### Inhibition of phosphodiesterase 4 expression, improvement in neurological deficits, and reduction in brain edema after subarachnoid hemorrhage by etazolate treatment

**Figure 3:**
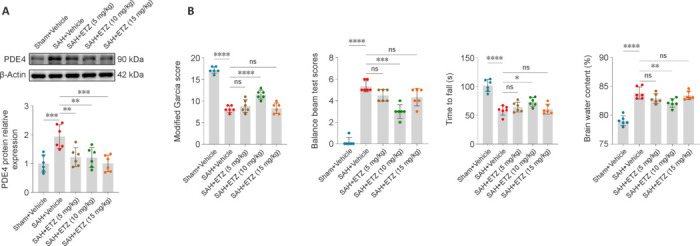
ETZ treatment inhibits PDE4 expression, improves neurological deficits, and reduces brain edema 24 hours after SAH. (A) Representative western blot band and relative protein expression of PDE4 in dosage course experiment. (B) The modified Garcia score, balance beam test, Rotarod test, and brain water content of all groups in the dosage course experiment. Data are presented as means ± SD; *n* = 6 per group. *P < 0.05, **P < 0.01, ***P < 0.001, ****P < 0.0001 (one-way analysis of variance with Tukey’s *post hoc* test or the Brown–Forsythe test). ETZ: Etazolate; ns: not significant; PDE4: phosphodiesterase 4; SAH: subarachnoid hemorrhage; Sham: sham surgery.

Next, ETZ was administered to investigate the effect of PDE4 on EBI, and to determine the optimal dose of ETZ. Western blot showed that all three doses of ETZ inhibited PDE4 expression (**Figure 3A**). The neurobehavioral experiments showed that 10 mg/kg ETZ improved the neurological deficits of mice, and the other two doses had a small effect, but did not reach statistical significance. When assessing the brain water content, 10 mg/kg ETZ alleviated brain edema after SAH, whereas the other two doses showed a tendency without reaching statistical significance (**Figure 3B**). According to the above experiments, 10 mg/kg ETZ was the most effective at ameliorating the neurological deficits and reducing brain edema in SAH mice, and inhibiting the PDE4 expression level. Therefore, a 10 mg/kg dose of ETZ was selected for the following experiments.

### Induced neuronal pyroptosis after subarachnoid hemorrhage via activation of phosphodiesterase 4

**Figure 4:**
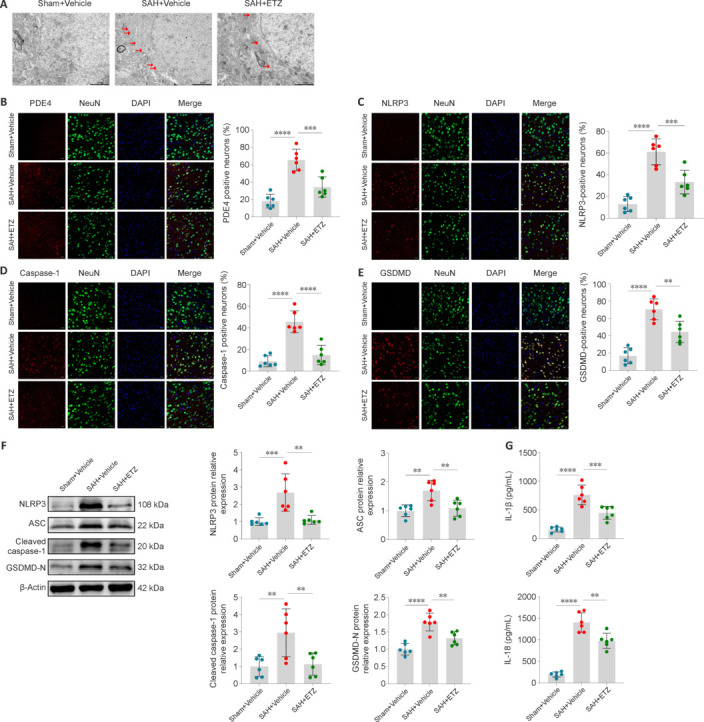
Activation of PDE4 induces neuronal pyroptosis 24 hours after SAH. (A) Representative TEM images of neurons. Red arrows: membrane pores. (B) Confocal IF staining of PDE4 (red) with neurons (NeuN, green) and proportion of PDE4-positive neurons. (C) Confocal IF staining of NLRP3 (red) with neurons (NeuN, green) and proportion of NLRP3-positive neurons. (D) Confocal IF staining of caspase-1 (red) with neurons (NeuN, green) and proportion of caspase-1-positive neurons. (E) Confocal IF staining of GSDMD (red) with neurons (NeuN, green) and proportion of GSDMD-positive neurons. Scale bars: 2 μm in A and 20 μm in B–E. (F) Representative western blot bands and relative protein expression of NLRP3, ASC, cleaved caspase-1, and GSDMD-N. (G) The levels of IL-1β and IL-18 in the cerebral cortex of mice detected by enzyme-linked immunosorbent assay. Data are presented as mean ± SD; *n* = 6 per group. **P < 0.01, ***P < 0.001, ****P < 0.0001 (one-way analysis of variance with Tukey’s *post hoc* test). ASC: Apoptosis-associated speck-like protein containing a caspase recruitment domain; Caspase-1: cysteinyl aspartate specific proteinase-1; ETZ: etazolate; GSDMD: gasderminD; IF: immunofluorescence; IL: interleukin; NLRP3: nucleotide-binding oligomerization domain-like receptor pyrin domain containing 3; PDE4: phosphodiesterase 4; SAH: subarachnoid hemorrhage; Sham: sham surgery; TEM: transmission electron microscopy.

After determining the optimal dose of ETZ, we further explored whether PDE4 mediates neuronal pyroptosis after SAH. One of the characteristics of pyroptosis is the formation of pores in the cell membrane, and thus TEM was used to observe changes in the neuronal membrane (Yu et al., 2021). The findings showed that pores formed in the neuronal membrane after SAH, and ETZ treatment mitigated this phenomenon (**Figure 4A**). Confocal IF was used to detect the expression and distribution of PDE4 along with pyroptosis-related molecules NLRP3, caspase-1, and gasdermin D (GSDMD). The results showed that the SAH group had an increase in PDE4 and the pyroptosis-related molecules in neurons compared with those in the Sham group. ETZ treatment reversed these SAH-induced changes (**Figure 4B–E**). Additionally, the protein levels of pyroptosis-related molecules NLRP3, ASC, cleaved caspase-1, and GSDMD-N in the SAH group were increased compared with those in the Sham group. Consistent with the IF results, ETZ treatment inhibited these increases (**Figure 4F**). ELISA was used to detect the secretion levels of pyroptosis-related inflammatory factors IL-1β and IL-18. ETZ treatment in the SAH + ETZ group reduced these inflammatory factors levels compared with the SAH + Vehicle group (**Figure 4G**).

**Figure 5:**
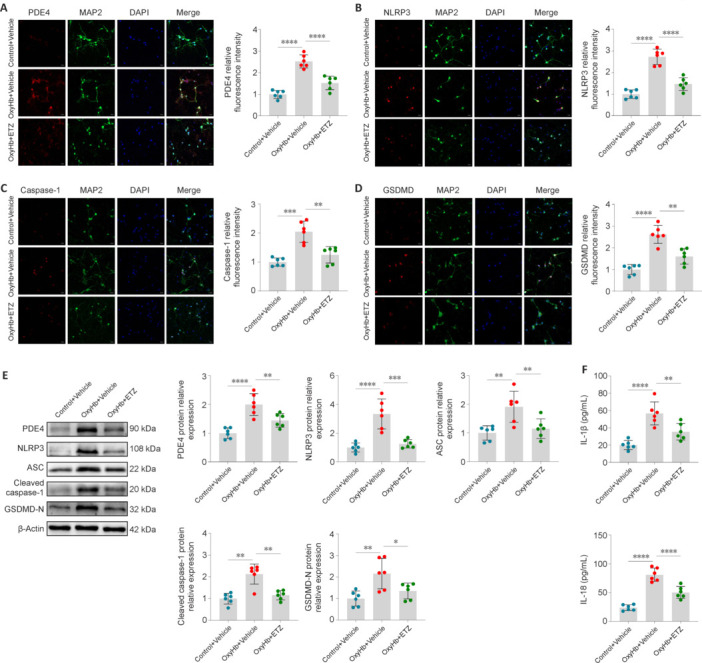
Activation of PDE4 induces pyroptosis of primary neurons 24 hours after OxyHb induction. (A) Confocal IF staining of PDE4 (red) with primary neurons (MAP2, green) and PDE4 relative fluorescence intensity. (B) Confocal IF staining of NLRP3 (red) with primary neurons (MAP2, green) and NLRP3 relative fluorescence intensity. (C) Confocal IF staining of caspase-1 (red) with primary neurons (MAP2, green) and caspase-1 relative fluorescence intensity. (D) Confocal IF staining of GSDMD (red) with primary neurons (MAP2, green) and GSDMD relative fluorescence intensity. Scale bars: 20 μm in A–D. (E) Representative western blot bands and relative protein expression of PDE4, NLRP3, ASC, cleaved caspase-1, and GSDMD-N. (F) The levels of IL-1β and IL-18 in primary neurons detected by enzyme-linked immunosorbent assay. Data are presented as mean ± SD; *n* = 6 per group. *P < 0.05, **P < 0.01, ***P < 0.001, ****P < 0.0001 (one-way analysis of variance with Tukey’s *post hoc* test or the Brown–Forsythe test). ASC: Apoptosis-associated speck-like protein containing a caspase recruitment domain; Caspase-1: cysteinyl aspartate specific proteinase-1; cells: primary neurons; ETZ: etazolate; GSDMD: gasderminD; IF: immunofluorescence; IL: interleukin; NLRP3: nucleotide-binding oligomerization domain-like receptor pyrin domain containing 3; OxyHb: oxyhemoglobin; PDE4: phosphodiesterase 4.

Similarly, the primary cortical neurons cultured *in vitro* were treated with 1 μM of ETZ to inhibit PDE4. IF and western blot showed that PDE4 and pyroptosis-related molecules expression levels in the OxyHb group were increased compared with those in the Control group. Consistent with the results of the *in vivo* experiment, ETZ treatment inhibited these increases (**Figure 5A–E**). Additionally, ETZ treatment decreased the levels of IL-1β and IL-18 in the OxyHb group (**Figure 5F**). These results indicated that pharmacological inhibition of PDE4 attenuated neuronal pyroptosis after SAH *in vivo* and *in vitro*.

### Induced neuronal injury after subarachnoid hemorrhage by activation of phosphodiesterase 4

**Figure 6:**
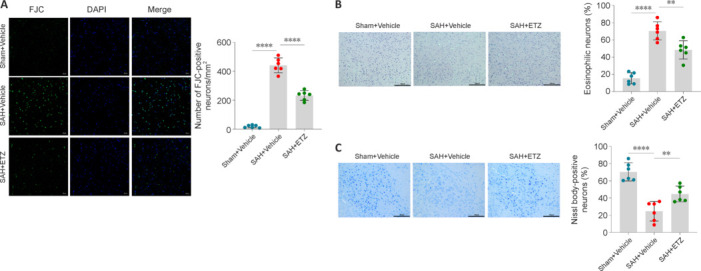
Activation of PDE4 induces neuronal injury 24 hours after SAH. (A) Representative FJC staining images of neurons and number of FJC-positive neurons (green). (B) Representative HE staining images of the cerebral cortex in mice and proportion of eosinophilic neurons (red). (C) Representative Nissl staining images of the cerebral cortex in mice and proportion of Nissl body–positive neurons (blue). Scale bars: 20 μm in A, 50 μm in B and C. Data are presented as mean ± SD; *n* = 6 per group. **P < 0.01, ****P < 0.0001 (one-way analysis of variance with Tukey’s *post hoc* test). Animals: C57BL/6J mice; ETZ: etazolate; FJC: Fluoro-Jade C; HE: hematoxylin-eosin; PDE4: phosphodiesterase 4; SAH: subarachnoid hemorrhage; Sham: sham surgery.

EBI can manifest as neuronal degeneration and morphological changes. Neuronal degeneration was detected by FJC staining. ETZ treatment reduced the number of FJC-positive neurons per mm^2^, indicating that it ameliorated neuronal degeneration in SAH mice (**Figure 6A**). Neuronal morphology was detected by HE and Nissl staining. The ETZ group had a decreased proportion of eosinophilic neurons compared with that in the SAH group, and the proportion of Nissl body-positive neurons was increased by ETZ treatment (**Figure 6B** and **C**). These results suggest that pharmacological inhibition of PDE4 attenuated neuronal degeneration and morphological damage, and thus improved EBI.

### Improvement in early brain injury and neuronal pyroptosis after subarachnoid hemorrhage by knockdown of endogenous phosphodiesterase 4

**Figure 7:**
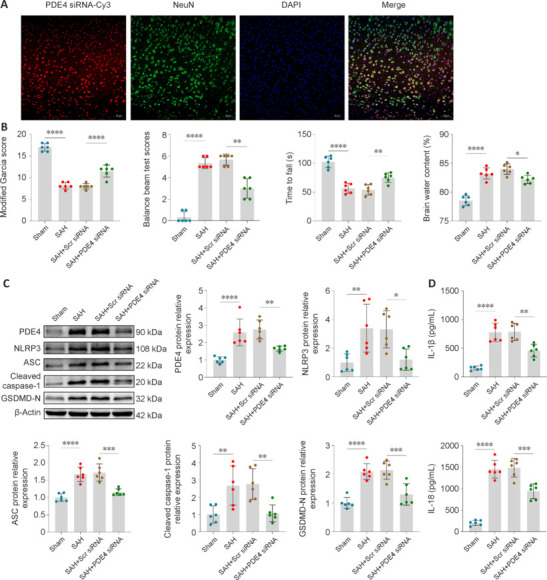
Knockdown of endogenous PDE4 improved EBI and neuronal pyroptosis 24 hours after SAH. (A) Confocal IF staining of Cy3-conjugated PDE4 siRNA (red) with neurons (NeuN, green) and proportion of PDE4 siRNA-positive neurons. Scale bars: 50 μm. (B) The modified Garcia score, balance beam test, Rotarod test, and brain water content of all groups. (C) Representative western blot bands and relative protein expression of PDE4, NLRP3, ASC, cleaved caspase-1, and GSDMD-N. (D) The levels of IL-1β and IL-18 in the cerebral cortex of mice detected by enzyme-linked immunosorbent assay. Data are presented as mean ± SD; *n* = 6 per group; *P < 0.05, **P < 0.01, ***P < 0.001, ****P < 0.0001 (one-way analysis of variance with Tukey’s *post hoc* test or the Brown–Forsythe test). ASC: Apoptosis-associated speck-like protein containing a caspase recruitment domain; Caspase-1: cysteinyl aspartate specific proteinase-1; EBI: early brain injury; GSDMD: gasderminD; IF: immunofluorescence; IL: interleukin; NLRP3: nucleotide-binding oligomerization domain-like receptor pyrin domain containing 3; PDE4: phosphodiesterase 4; SAH: subarachnoid hemorrhage; Scr: scrambled; Sham: sham surgery; siRNA: small interfering RNA.

The pharmacological effects of ETZ may involve other processes, and therefore we next investigated whether modulation of PDE4 at the genetic level would produce the same anti-pyroptosis and EBI ameliorating effects as ETZ treatment. PDE4 siRNA was i.c.v. injected to genetically inhibit PDE4. Confocal IF showed that Cy3-conjugated PDE4 siRNA was costained with neurons and stably expressed, with an average transfection efficiency of 85.33% (**Figure 7A**). Compared with the SAH + Scr siRNA group, the genetic suppression of PDE4 reduced the neurological deficits and alleviated the brain edema in mice (**Figure 7B**). Administering PDE4 siRNA inhibited the increase in the expression of PDE4 and pyroptosis-related molecules (**Figure 7C**). Additionally, the levels of pyroptosis-related inflammatory factors were clearly reduced owing to the genetic knockdown of PDE4 (**Figure 7D**). These results showed that PDE4 knockdown and ETZ treatment had the same effect, which validated the specific mechanism of action of anti-pyroptosis in ETZ, and indicated that both the pharmacological and genetic inhibition of PDE4 improved EBI by attenuating neuronal pyroptosis.

### Blocking of nuclear factor kappa-B pathway after subarachnoid hemorrhage by phosphodiesterase 4 inhibition

**Figure 8:**
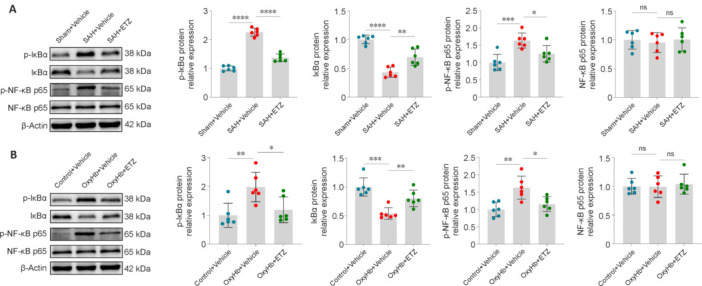
PDE4 inhibition blocks the NF-κB pathway 24 hours after SAH. (A, B) Representative western blot bands and relative protein expression of p-IκBα, IκBα, p-NF-κB p65, and NF-κB p65 in the cerebral cortex of mice (A) and primary neurons (B). Data are presented as mean ± SD; *n* = 6 per group. *P < 0.05, **P < 0.01, ***P < 0.001, ****P < 0.0001 (one-way analysis of variance with Tukey’s *post hoc* test or the Brown–Forsythe test). ETZ: Etazolate; IκBα: inhibitor kappa B alpha; NF-κB: nuclear factor kappa-B; ns: not significant; OxyHb: oxyhemoglobin; PDE4: phosphodiesterase 4; SAH: subarachnoid hemorrhage; Sham: sham surgery.

The NF-κB signaling pathway is essential for the activation of the NLRP3 inflammasome (Lamkanfi and Dixit, 2014). To determine whether ETZ treatment affects NF-κB signaling after SAH, the expression levels of related proteins from mice and primary neurons were detected by western blot. The results suggested that in the *in vivo* and *in vitro* SAH models, ETZ treatment reduced IκBα phosphorylation and increased its basal level. It also reduced NF-κB p65 phosphorylation and maintained its basal level (**Figure 8A** and **B**). These findings suggest that inhibition of PDE4 suppresses NF-κB signaling after SAH.

### Improvement in lysosomal function after subarachnoid hemorrhage by phosphodiesterase 4 inhibition

**Figure 9:**
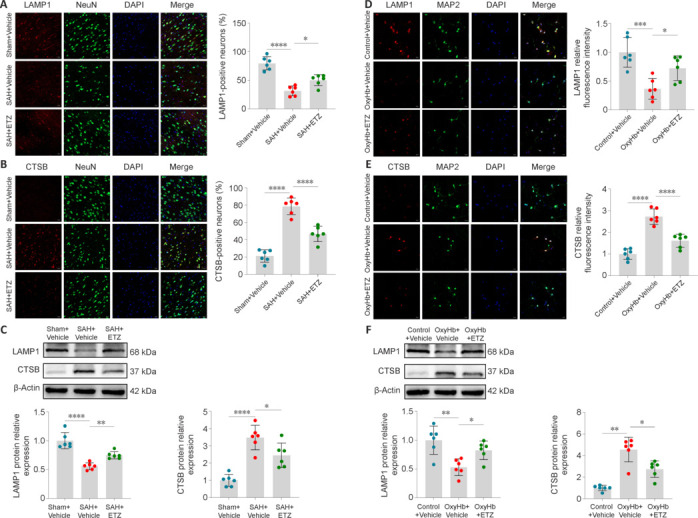
PDE4 inhibition improves lysosomal function 24 hours after SAH. (A) Confocal IF staining of LAMP1 (red) with neurons (NeuN, green) and proportion of LAMP1-positive neurons. (B) Confocal IF staining of CTSB (red) with neurons (NeuN, green) and proportion of CTSB-positive neurons. (C) Representative western blot bands and relative protein expression of LAMP1 and CTSB in the cerebral cortex of mice. (D) Confocal IF staining of LAMP1 (red) with primary neurons (MAP2, green) and LAMP1 relative fluorescence intensity. (E) Confocal IF staining of CTSB (red) with primary neurons (MAP2, green) and CTSB relative fluorescence intensity. Scale bars: 20 μm in A, B, D, and E. (F) Representative western blot bands and relative protein expression of LAMP1 and CTSB in primary neurons. Data are presented as mean ± SD; *n* = 6 per group. *P < 0.05, **P < 0.01, ***P < 0.001, ****P < 0.0001 (one-way analysis of variance with Tukey’s *post hoc* test or the Brown–Forsythe test). CTSB: Cathepsin B; ETZ: etazolate; IF: immunofluorescence; LAMP1: lysosomal associated membrane protein 1; OxyHb: oxyhemoglobin; PDE4: phosphodiesterase 4; SAH: subarachnoid hemorrhage; Sham: sham surgery.

Lysosomal dysfunction can induce the activation of the NLRP3 inflammasome (Yang et al., 2019). It is usually characterized by increased lysosomal membrane permeability or lysosomal rupture, both of which lead to decreased expression of lysosomal membrane marker LAMP1 and the release of lysosomal contents, such as CTSB, into the cytoplasm and their activation. Confocal IF and western blot showed that LAMP1 expression in cortical neurons decreased after SAH, whereas that of CTSB increased compared with those in the Sham group. Inhibition of PDE4 reversed these changes (**Figure 9A–C**). Consistent results were obtained *in vitro* (**Figure 9D–F**). These findings indicated that PDE4 inhibition reduced CTSB release and improved lysosome function by stabilizing lysosomal membranes or inhibiting lysosome rupture.

### Improvement in mitochondrial function after subarachnoid hemorrhage by phosphodiesterase 4 inhibition

**Figure 10:**
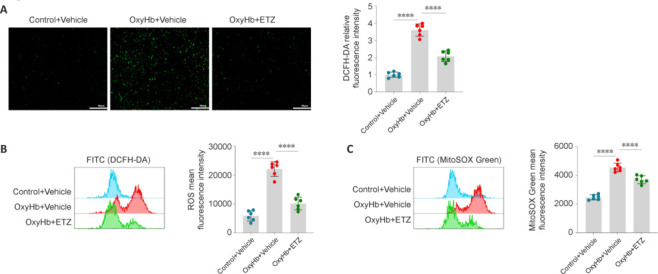
PDE4 inhibition improves mitochondrial function 24 hours after SAH. (A) Total intracellular ROS levels detected by IF of DCFH-DA fluorescent probe (green) and DCFH-DA relative fluorescence intensity in primary neurons. Scale bars: 50 μm. (B) Total intracellular ROS levels detected by flow cytometry of DCFH-DA fluorescent probe and DCFH-DA mean fluorescence intensity in primary neurons. (C) Mitochondrial-specific ROS levels detected by flow cytometry of MitoSOX Green fluorescent probe and MitoSOX Green mean fluorescence intensity in primary neurons. Data are presented as mean ± SD; *n* = 6 per group. ****P < 0.0001 (one-way analysis of variance with Tukey’s *post hoc* test). ETZ: Etazolate; IF: immunofluorescence; OxyHb: oxyhemoglobin; PDE4: phosphodiesterase 4; ROS: reactive oxygen species.

Mitochondrial dysfunction can induce the activation of the NLRP3 inflammasome by producing excess ROS (Yang et al., 2019). ROS were labeled by a DCFH-DA fluorescent probe and then detected by IF and flow cytometry. ETZ treatment reduced the ROS levels in OxyHb-induced primary neurons (**Figure 10A** and **B**). This approach measured the total intracellular ROS, which contained multiple sources. Therefore, we next used MitoSOX mitochondrial superoxide to detect mitochondrion-specific ROS to improve specificity. The mitochondrion-specific ROS levels in primary neurons increased after OxyHb induction, which was reversed by ETZ treatment (**Figure 10C**). These findings suggest that PDE4 inhibition improved mitochondrial function and attenuated ROS production.

### Reversal of the neuroprotective effect of phosphodiesterase 4 inhibition by specific activation of nucleotide-binding oligomerization domain-like receptor pyrin domain containing 3

**Figure 11:**
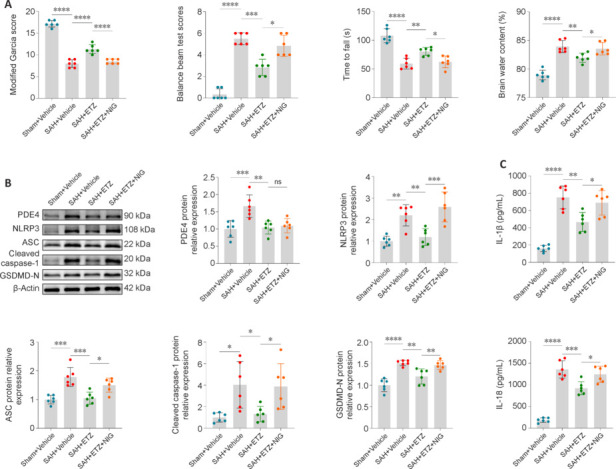
Specific activation of NLRP3 reversed the neuroprotective effect of PDE4 inhibition 24 hours after SAH. (A) The modified Garcia score, balance beam test, Rotarod test, and brain water content of all groups. (B) Representative western blot bands and relative protein expression of PDE4, NLRP3, ASC, cleaved caspase-1, and GSDMD-N in the cerebral cortex of mice. (C) The levels of IL-1β and IL-18 in the cerebral cortex of mice detected by enzyme-linked immunosorbent assay. Data are presented as mean ± SD; *n* = 6 per group; *P < 0.05, **P < 0.01, ***P < 0.001, ****P < 0.0001 (one-way analysis of variance with Tukey’s *post hoc* test or the Brown–Forsythe test). ASC: Apoptosis-associated speck-like protein containing a caspase recruitment domain; Caspase-1: cysteinyl aspartate specific proteinase-1; ETZ: etazolate; GSDMD: gasderminD; IL: interleukin; NIG: nigericin; NLRP3: nucleotide-binding oligomerization domain-like receptor pyrin domain containing 3; ns: not significant; PDE4: phosphodiesterase 4; SAH: subarachnoid hemorrhage; Sham: sham surgery.

To investigate whether PDE4 mediates EBI and neuronal pyroptosis by regulating NLRP3, and the relationship between PDE4 and NLRP3 in the signaling pathway, the NLRP3-specific agonist NIG was administrated. Neurobehavioral experiments and brain water content measurement showed that NLRP3 activation in the SAH + ETZ + NIG group reversed the improvement effects of ETZ on neurological dysfunction and brain edema in SAH mice compared with the SAH + ETZ group (**Figure 11A**). Western blot and ELISA showed that NIG reversed the effects of ETZ on the reduction in pyroptosis-related protein and inflammatory factor levels. However, it did not affect the inhibition of ETZ on PDE4 (**Figure 11B** and **C**). These findings indicated that NLRP3 activation reversed the attenuating effect of ETZ on neuronal pyroptosis in SAH mice, and NLRP3 was downstream of PDE4 in the signaling pathway.

## Discussion

This study demonstrated that PDE4 peaked 24 hours after SAH, and it was localized to neurons only. The results suggested that pharmacological inhibition of PDE4 by ETZ and gene inhibition of PDE4 by siRNA improved EBI by reducing neuronal pyroptosis. The mechanism may be that PDE4 activated the NF-κB pathway and caused lysosomal and mitochondrial dysfunction. These three factors then promoted NLRP3 activation and induced neuronal pyroptosis. An NLRP3 agonist reversed the neuroprotective effect of PDE4 inhibition.

At present, early pathophysiological changes secondary to SAH are recognized as the main cause of mortality in patients. EBI includes a series of pathological events, such as nerve cell death, destruction of the blood–brain barrier, brain edema, acute cerebrovascular spasm, and microvascular dysfunction (Cahill et al., 2006; Fan et al., 2021). Elucidating the pathogenesis of EBI after SAH could be conducive to formulating early treatment strategies after SAH, alleviate the severity of EBI, slow down or even block the development of secondary brain injury or reduce its severity, and improve the prognosis of SAH patients (Hu et al., 2021; Wang et al., 2024).

Traditionally, nerve cell apoptosis has been considered the main pathological mechanism of EBI, and numerous studies on nerve cell apoptosis after SAH have been conducted over the past decades (Tian et al., 2023). However, clinical translation found that targeted therapy for apoptosis did not completely improve patient outcomes, and therefore the overall research direction gradually shifted to other forms of cell death (Zhang et al., 2022). Recently, studies have shown that pyroptosis occurs in nerve cells after SAH, and preventing pyroptosis can greatly improve the outcome of SAH (Yuan et al., 2020; Fang et al., 2022; Liu et al., 2023). These findings indicate that pyroptosis plays an important role in the pathophysiological progression of EBI after SAH. The mechanism by which pyroptosis plays a role may be explained as follows. When cells are stimulated, intracellular inflammasomes form protein complexes for intracellular signal transduction under the action of various signals. Subsequently, caspase-1 is activated and it cleaves GSDMD to release the N-terminal domain to form active GSDMD-N. This pore-forming activity damages the cell membrane integrity, destroys the cellular and extracellular osmotic balance, disrupts the electrolyte balance, causes cell swelling and rupture, releases large amounts of inflammatory factors and cellular contents, and recruits immune cells to further exacerbate and worsen the inflammatory response (Yu et al., 2021; Rao et al., 2022). In the present study, neurons in SAH mice underwent obvious pyroptosis and damage, with pores forming on the cell membrane and a marked inflammatory response. Inhibiting pyroptosis reversed these phenomena. This finding supports the idea of improving pyroptosis in EBI therapy.

Pyroptosis mediated by inflammasomes is considered the classic pathway of pyroptosis, in which NLRP3 is the most characteristic inflammasome and has been well studied (Huang et al., 2021). NLRP3 activation requires two signals: initially, there is a rise in NLRP3 gene transcription, reliant on the activation of pattern recognition receptors to trigger the NF-κB pathway; the second is the activation of NLRP3 stimulants, such as endogenous danger-associated molecules, including CTSB and ROS (Schroder and Tschopp, 2010; Huang et al., 2021). This study and our previous research showed that NLRP3 is activated after SAH (Zhao et al., 2017). Additionally, SAH activated the NF-κB pathway, accompanied by lysosomal and mitochondrial dysfunction, which resulted in CTSB activation and ROS accumulation within neurons.

PDE4 is an enzyme with a high affinity for its substrate, specifically targeting the hydrolysis of cAMP and rendering it biologically inactive. This enzymatic degradation is the sole mechanism by which cAMP can be effectively degraded (Fertig and Baillie, 2018). cAMP is usually a second messenger that participates in regulating intracellular processes after receiving various extracellular signals and maintains the physiological activity of cells (Houslay, 2010; Gancedo, 2013). Therefore, when the body is injured or stressed, leading to changes in PDE4 expression, the balance of cAMP hydrolysis is disrupted, resulting in changes in cell physiological activity, which may lead to cell death in severe cases (Gao et al., 2022). PDE4 has been shown to mediate nervous system injury in Alzheimer’s disease, Parkinson’s disease, and depression, but the mechanisms remain unclear (Vagena et al., 2019; Zhong et al., 2019; Meng et al., 2022). Li et al. (2019) reported that PDE4 inhibition alleviated colitis in mice by blocking the NF-κB pathway. Hong et al. reported that blockade of PDE4 in HeLa cells stabilized lysosomal function and reduced lysosomal enzyme activity (Hong et al., 2017). Liu et al. (2022) reported that in an *in vitro* oxidative stress model, PDE4 inhibition improved mitochondrial function in neurons and suppressed ROS generation. Together, the above findings regarding the NLRP3 activation signal suggest that PDE4 may act as an important upstream molecule in cell pyroptosis by affecting the NF-κB pathway, lysosomal function, and mitochondrial function, thereby influencing the activation of NLRP3. Thus, PDE4 may activate the NLRP3 inflammasome indirectly after SAH. The findings of the present study support this idea. Activation of NLRP3 did not affect PDE4 expression, suggesting that NLRP3 acts in the downstream signaling of PDE4.

Currently, the main strategies for regulating PDE4 are focused on the development of its specific inhibitors. Clinical studies of specific PDE4 inhibitors have included various disease areas, such as central nervous system disorders, respiratory system diseases, autoimmune diseases, and skin diseases (Liu et al., 2022). The use of PDE4 inhibitors rolipram and roflumilast in experimental SAH improved the prognosis of rats (Wu et al., 2017; Li et al., 2018). ETZ is a pyrazolo pyridine, and previous studies have concentrated on its use in treating Alzheimer’s disease, depression, and anxiety (Jindal et al., 2015; Alzoubi et al., 2017; Chen et al., 2020). ETZ has a very small molecular weight (289.33 g/mol), which allows it to easily pass through the blood–brain barrier and exert its neuropharmacological effects (Jindal et al., 2017). In terms of mechanism, ETZ is a selective γ-aminobutyric acid-A receptor modulator. In cortical neuron cells of rats, ETZ stimulates the alpha-secretase pathway to induce the generation of amyloid precursor protein, and it inhibits the neurotoxicity of amyloid-β in cortical neurons by regulating γ-aminobutyric acid-A receptors (Marcade et al., 2008). Furthermore, ETZ is a specific PDE4 inhibitor that produces antidepressive and antianxiety effects in animal models by inhibiting PDE4 (Jindal et al., 2012, 2013). Siopi et al. (2013) showed that ETZ reduced neuroinflammation in mouse brain injury and provided sustained neuroprotection. Therefore, ETZ was used in the present study. The findings indicate that ETZ improved neuronal pyroptosis and EBI after SAH by inhibiting PDE4, suggesting, to some extent, that ETZ is a potential therapeutic agent for SAH.

This study had several limitations. The animals used in this study were all male because female mice have periodic hormonal fluctuations that can significantly affect the results of neuroscience experiments. But single-sex experiments inevitably lead to some degree of bias in the results. In the future, more scientific animal sex management methods need to be developed in neuroscience-related experiments to guide preclinical research (Dinh et al., 2024). The mechanism underlying the involvement of PDE4 in neuronal pyroptosis was not explored in depth, and only NLRP3 was regulated in the downstream pathway, without the regulation of the NF-κB pathway, lysosomes, or mitochondrial function. These are worthy of further research in the future. Although ETZ significantly inhibited the expression of PDE4, a transgenic PDE4 knockout animal model is needed to validate the findings.

In conclusion, PDE4 promoted NLRP3 activation, induced neuronal pyroptosis, and participated in EBI after experimental SAH. Inhibition of PDE4 improved EBI by reducing neuronal pyroptosis. PDE4 may be a potential therapeutic molecular target for SAH.

## Data Availability

*No additional data are available*.
